# SurveyNet: A Unified Deep Learning Framework for OCR and OMR-Based Survey Digitization

**DOI:** 10.3390/jimaging12040175

**Published:** 2026-04-17

**Authors:** Rubi Quiñones, Sreeja Cheekireddy, Eren Gultepe

**Affiliations:** Department of Computer Science, Southern Illinois University Edwardsville, Edwardsville, IL 62026, USA; scheeki@siue.edu (S.C.); egultep@siue.edu (E.G.)

**Keywords:** survey digitization, optical character recognition (OCR), optical mark recognition (OMR), deep learning, convolutional neural networks (CNN), handwritten digit recognition, form processing, data automation, image classification, real-world dataset

## Abstract

Manual survey data entry remains a bottleneck in large-scale research, marketing, and public policy, where survey sheets are still widely used due to accessibility and high response rates. Despite the progress in Optical Character Recognition (OCR) and Optical Mark Recognition (OMR), existing systems treat these tasks separately and are typically tailored to clean, standardized forms, making them unreliable for real-world survey sheets with diverse markings and handwritten inputs. These limitations hinder automation and introduce significant error rates in data transcription. To address this, we propose SurveyNet, a unified deep learning framework that combines OCR and OMR capabilities to automatically digitize complex survey responses within a single model. SurveyNet processes both handwritten digits and a wide variety of mark types including ticks, circles, and crosses across multiple question formats. We also introduce SurveySet, a novel dataset comprising 135 real-world survey forms annotated across four key response types. Experimental results demonstrate that SurveyNet achieves between 50% and 97% classification accuracy across tasks, with strong performance even on small and imbalanced datasets. This framework offers a scalable solution for streamlining survey digitization workflows, reducing manual errors, and enabling timely analysis in domains ranging from consumer research to public health and education.

## 1. Introduction

Surveys are a foundational tool in research and data-driven decision-making, used widely in sectors such as healthcare [1,2], education [3], marketing [4,5], and social sciences [6]. Despite the increasing adoption of digital survey platforms [7], paper-based surveys remain common due to their accessibility, ease of distribution, and higher response rates; particularly in populations with limited digital infrastructure or lower computer literacy [8]. In contexts such as in-person interviews, community assessments, or outreach in underserved areas, physical survey forms remain a practical and often necessary data collection method.

However, the process of manually transcribing survey responses into digital systems introduces significant challenges. Manual data entry is time-consuming, labor-intensive, and prone to human error, particularly when dealing with large-scale studies or forms with mixed response types [8]. Traditional digitization techniques rely on Optical Mark Recognition (OMR) for detecting filled-in choices [9] and Optical Character Recognition (OCR) for interpreting text or digits [10]. These approaches are often applied in isolation and are typically calibrated for highly structured documents, such as standardized test sheets with clearly defined bubbles [9,11].

In real-world survey settings, however, the variability in how respondents mark their answers complicates this process. Responses may be indicated by circles, crosses, tick marks, scribbles, or even partially filled boxes, marking styles that fall outside the rigid expectations of classic OMR systems [12,13]. Similarly, handwritten numerical responses are often inconsistent in spacing, scale, and legibility, making OCR less reliable without substantial preprocessing [9]. Most existing systems either fail to generalize to these complexities or require custom rule-based configurations that do not scale across datasets or domains [9,14]. In contrast, SurveyNet is trained on a diverse range of realistic marking and handwriting variations within a fixed survey template, enabling the model to learn shared representations across heterogeneous response categories rather than relying on brittle, rule-based heuristics. While this design reduces dependence on handcrafted rules, the present study does not claim uniform generalization across all task types or layouts, and performance is observed to be task-dependent. By capturing these variations within the learning process, SurveyNet reduces reliance on rigid, rule-based heuristics and supports adaptation to moderate marking style variation within the surveyed template, rather than assuming uniform generalization across all response types or layouts.

Recent advances in computer vision and deep learning, particularly convolutional neural networks (CNNs), have opened new opportunities for document analysis. CNN-based models [15,16,17] have shown state-of-the-art performance in handwritten text recognition, form understanding, and mark detection across a variety of applications. For example, deep learning frameworks have been used to extract features from scanned forms and classify responses with high accuracy, especially in controlled environments [18,19]. More recent OCR methods leverage advanced sequence modeling and attention-based architectures, such as Convolutional Recurrent Neural Networks (CRNNs), Long Short-Term Memory (LSTM), and Connectionist Temporal Classification (CTC), to capture complex contextual relationships within and across text sequences. These approaches learn hierarchical dependencies between characters, words, and spatial structures, allowing the model to account for irregular layouts, cursive handwriting, and noisy document conditions. Such methods have demonstrated improved recognition accuracy on unstructured or handwritten text in recent studies [20,21,22]. However, these systems tend to focus on either OCR or OMR in isolation and often require large, well-annotated datasets for training. The lack of a unified approach that can handle both OCR and OMR tasks, especially in noisy, real-world survey forms, remains a critical limitation. Furthermore, the absence of publicly available, annotated datasets that reflect the diversity of real-world survey responses limits progress in this area.

To address these challenges, we propose SurveyNet, a deep learning framework that unifies OCR and OMR tasks within a single CNN-based pipeline, enabling automation of survey response digitization. SurveyNet is designed to process both handwritten digits and a wide range of mark types including ticks, circles, and crosses, without relying on form templates or domain-specific rules. To support this framework, we also introduce SurveySet, a new dataset comprising 135 real-world survey forms, categorized into four task types: numerical scales, binary options, complex multiple-choice questions, and handwritten numeric answers. These forms reflect realistic conditions including noise, variable handwriting, and inconsistent marking styles. The dataset also contains naturally occurring distortions such as minor blurring, scanner-induced skew, uneven lighting, and faint or partial strokes, which arise in practical digitization workflows. Our contributions are as follows:

1. SurveyNet: A unified deep learning framework that integrates OCR and OMR to digitize diverse survey responses in a single model.
2. SurveySet: A novel dataset of 135 annotated survey forms with realistic complexity and heterogeneous response styles.
3. Benchmark study: A comparison against baseline OCR and OMR models.
4. Efficacy study: An analysis of task-dependent performance across varying mark types and response formats, showing that SurveyNet achieves up to 97% classification accuracy on well-structured response categories while exhibiting substantial variability under imbalanced and low-data conditions.

## 2. Related Works

This section reviews prior work in OCR and OMR, focusing on their roles in automating data extraction from physical forms. We compare traditional rule-based approaches with modern learning-based models and highlight their respective strengths and limitations. Finally, we identify gaps in unified OCR–OMR systems and motivate the development of our proposed framework and dataset.

### 2.1. Optical Character Recognition Methods

*OCR* enables the conversion of scanned (printed or handwritten) text into machine-readable digital text. Historically, OCR systems used rule-based approaches such as template matching and pattern recognition. Template matching compares characters in scanned images against a fixed set of stored templates, but it struggles with unseen fonts, distortions, or handwriting [23,24,25]. Pattern recognition methods attempted to classify characters based on their shapes and structures, but lacked adaptability to stylistic variation [26,27]. Traditional OCR systems also used histogram-based feature extraction to analyze character intensity profiles, shapes, and contours [28,29,30]. These approaches were limited by their sensitivity to noise, variations in resolution, and character alignment, especially in handwritten or low-quality forms [31].

The adoption of CNNs has significantly advanced the field of OCR. CNNs have the capacity to learn spatial hierarchies from raw pixel data, reducing the need for manual feature engineering [26]. One notable approach, TextBoxes by Liao et al. [32], achieved 84% accuracy on scene text by combining a fully convolutional network with a text-specific bounding box regression. Similarly, the Tesseract OCR engine, which incorporates Long Short-Term Memory (LSTM) layers in its newer versions, demonstrated considerable improvements over rule-based engines for printed text detection in scanned documents [33].

Beyond printed text, CNNs have also proven effective in handwritten digit and character recognition. Vaidya et al. [34] achieved high accuracy on handwritten English alphabets and digits using a deep CNN architecture. Shetty and Sharma [35] proposed an ensemble CNN model that outperformed single-network baselines, achieving over 91% accuracy on structured printed datasets. Pashine et al. [36] used a handwritten dataset of 70,000 digits and achieved a peak accuracy of 99% using a five-layer CNN. These results highlight the robustness of CNNs for OCR tasks across varying input complexities.

However, despite strong performance on clean and segmented benchmarks, these models often fail in settings where handwritten digits appear adjacent to irregular markings, or when form layouts vary unpredictably. Existing OCR frameworks [27] typically assume isolated character zones and cannot dynamically adapt to structural noise, layout shifts, or overlapping symbols; all of which are common in real-world survey documents. They also lack mechanisms to reason over multiple cues (e.g., handwriting style, positional layout, surrounding context), which are critical for interpreting ambiguous inputs.

Recent work in other visual domains has tackled similar challenges by integrating multiple spatial and contextual features into the learning process. For instance, co-segmentation techniques developed by Quiñones et al. [37,38] leverage shared attention across regions to interpret weakly segmented or occluded structures. Such multi-feature reasoning is directly relevant to survey digitization, where digits may bleed into response areas or exhibit partial markings. These techniques suggest that OCR accuracy could improve if models were adapted to co-learn handwriting and structural layout features in tandem.

### 2.2. Optical Mark Recognition

Traditional OMR systems are typically deployed in structured environments such as standardized tests. These systems rely on pixel intensity thresholding within predefined mark zones, identifying a filled bubble when darkness exceeds a certain level [39]. While computationally efficient, they are rigid in their requirements: marks must be precisely placed and uniformly filled. Marks such as ticks, slashes, or partial shading are often misclassified. In addition, these systems are sensitive to poor scan quality, alignment errors, and stray marks, conditions that are common in field-collected surveys [14].

Deep learning has brought enhanced flexibility and accuracy to OMR systems. CNN-based approaches learn to detect varied mark types such as crosses, circles, and ticks without relying on rigid formatting. Mondal et al. [12] introduced OMRNet, which achieved 95% accuracy in identifying box-style marks using a lightweight CNN trained on exam sheet datasets. Loke et al. [40] built a software-based OMR model capable of interpreting both bubble and box fields, achieving 99% accuracy on structured forms.

These methods demonstrate strong performance in controlled settings, but their generalizability to unconstrained formats remains limited. Afifi et al. [13] evaluated a CNN-based OMR model on a dataset containing complex marking styles and overlapping annotations. Their model achieved over 90% accuracy in clean segments but saw a significant drop when processing marks overlaid with text. Such limitations highlight the difficulty of generalizing across real-world documents without layout consistency.

Recent work has attempted to close this gap. For instance, Alam et al. [41] used CNNs with image augmentation techniques to improve robustness against varying mark styles and paper quality, resulting in a 7% accuracy gain compared to traditional OMR pipelines. However, even these methods typically exclude adjacent textual content, making them poorly suited for mixed-format surveys. There remains a need for models that can jointly process symbolic and textual responses in a unified framework.

Additionally, many deep OMR frameworks treat response marks as isolated visual tokens, ignoring spatial patterns, question structures, or handwriting interference. In high-density regions, this leads to misclassification of ambiguous marks or double-reads of overlapping inputs. Methods such as GCNet [42], demonstrate how occlusion-aware modeling and correction strategies can substantially improve performance in visually complex scenes. GCNet explicitly incorporates a correction factor to recover from missed or occluded instances, a technique that could be adapted to OMR by recognizing when a respondent’s mark style deviates from expected templates or overlaps with neighboring text. These insights open the door to more context-sensitive OMR models that treat response zones as structured, not independent, features.

Despite advances in both OCR and OMR, very few systems attempt to address both tasks simultaneously within a single recognition framework. Most practical systems rely on custom pipelines that separately handle textual regions with OCR and mark regions with OMR, often requiring manual layout parsing and heuristic rules to decide which module to apply. Such siloed workflows introduce brittleness, since small errors in routing or ambiguous cases, for example, a handwritten digit written close to a partially filled box can cause misclassification and propagate downstream. Moreover, heuristic routing must be hand-tuned for each survey template, limiting scalability when forms vary in design. To address these limitations, we propose SurveyNet, a unified deep learning model that learns shared representations for both OCR and OMR tasks within a single architecture. While minimal layout information is still needed to locate answer regions, once identified, SurveyNet eliminates the need for task-specific pipelines or handcrafted rules.

This design enables the model to handle diverse response types: including ticks, crosses, and handwritten digits, within a single trainable recognition framework, reducing dependence on brittle, task-specific rule-based integration. Rather than claiming uniformly high accuracy across all tasks, we emphasize that SurveyNet removes the need for separate OCR and OMR pipelines within a fixed survey template, allowing performance to be driven primarily by data quality and response format rather than handcrafted routing logic.

### 2.3. Existing Survey Datasets

A number of datasets have significantly contributed to advancing the fields of OCR and OMR. These resources have supported model benchmarking, enabled progress in handwritten and marked response recognition, and fostered broad innovation in form understanding. A summary comparison of these datasets is shown in Table 1, highlighting their structure, mark types, and character support. The table also considers layout variability which is the extent to which form structure and element positioning change between samples, and form context, which indicates whether a dataset includes surrounding content such as labels, question prompts, or page layout cues. However, most existing datasets were designed to support one modality, either textual or mark-based recognition, and often under controlled, uniform conditions. As a result, there remains a need for datasets that reflect the mixed-input, layout-diverse nature of real-world survey forms.

For OCR, the MNIST dataset [43] has been instrumental in the development of handwritten digit classifiers. Its simplicity, balanced classes, and accessibility have made it a foundational benchmark. Likewise, the IAM dataset [44] provides valuable handwritten English text in line and word formats and has supported extensive work in document transcription and handwriting analysis. However, neither dataset is form-based, and both lack contextual layout or mixed-type responses.

In the domain of OMR, datasets such as those from Afifi et al. [13] and the OMRNet study [12] have enabled the development of models capable of interpreting filled bubbles, boxes, and other structured response formats. These datasets provide reliable ground truth for mark detection and perform well in exam or evaluation contexts where templates and uniform mark styles are expected. Their contributions to template-based OMR system performance are substantial.

**Figure 1:**
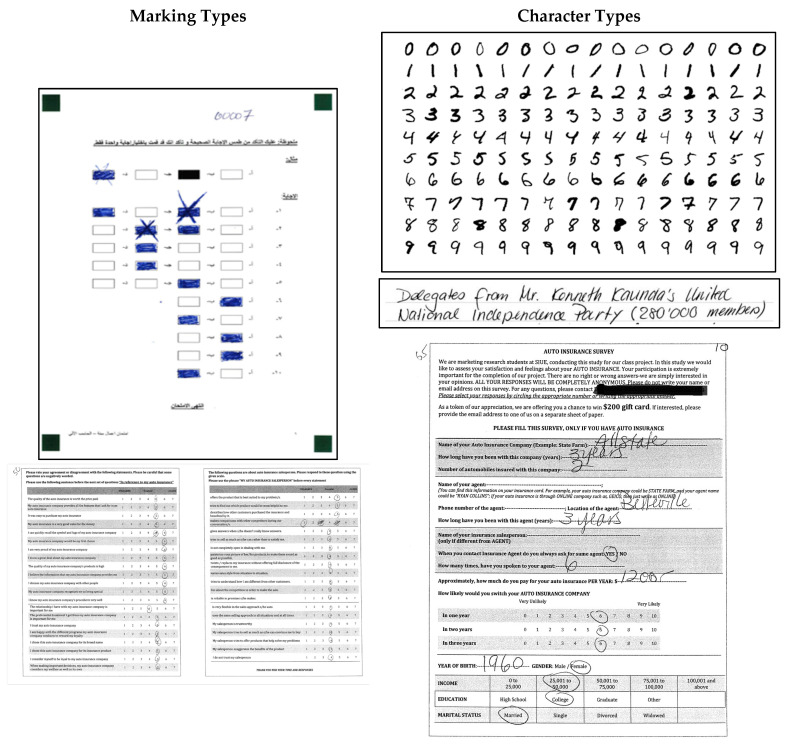
Sample images from selected OCR and OMR datasets. The left column illustrates common marking styles typically found in OMR datasets, while the right column shows examples of handwritten characters used in OCR tasks. The bottom row exclusively displays samples from SurveySet, demonstrating its coverage of both marking styles and handwritten input.

At the same time, these datasets are typically limited to specific formats such as multiple-choice fields, aligned forms, and clean mark boundaries. They do not include free-form handwritten responses or irregular markings such as partial circles, tick marks, or text-mark overlap. Moreover, they are not designed for tasks requiring the joint recognition of characters and markings within the same visual field, a common scenario in survey forms where users may circle options and also write a numeric value nearby. To see sample images from the mentioned datasets, refer to Figure 1.

To complement this prior work and expand the design space for document digitization models, we introduce SurveySet, a new dataset of 135 annotated customer survey forms. SurveySet is built from real-world documents and includes a mix of handwritten digits and informal marks across multiple formats: numerical scales, binary options, overlaid text-mark fields, and open numeric responses. It incorporates both character and mark annotations along with question-level context, enabling a unified evaluation of OCR–OMR systems. SurveySet was constructed to capture meaningful variability in respondent behavior: including handwriting diversity, inconsistent marking styles, and scan-induced noise, while maintaining a fixed questionnaire template in order to isolate and evaluate the feasibility of unified OCR–OMR recognition across core response types. By combining layout diversity, varied mark styles, and handwritten input, SurveySet offers a valuable resource for developing models that are robust to the complexities of real-world survey digitization while building on the strengths of existing datasets.

## 3. Proposed Framework

**Figure 2:**
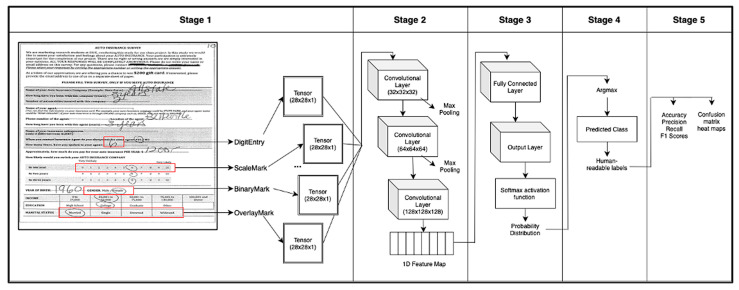
Overview of the 5 stage SurveyNet pipeline for unified survey response classification.

This section introduces SurveyNet, our unified deep learning framework for jointly recognizing handwritten characters and marked responses in survey forms. SurveyNet is designed to process real-world inputs with diverse layouts, overlapping mark and character regions, and informal response styles. In this work, the term unified refers specifically to the recognition stage, where multiple response types are handled within a single model, rather than implying a fully end-to-end pipeline. The system does not perform autonomous form parsing; instead, it operates on pre-identified answer regions. These regions are obtained through a semi-automated, layout-specific cropping process rather than a fully autonomous detection module. We describe the core model architecture, training pipeline, and the strategy for multi-modal classification. Figure 2 provides a visual overview of the proposed system.

### 3.1. Problem Definition

Let $D={\left\{(x_{i},y_{i})\right\}}_{i=1}^{N}$ represent a dataset of N grayscale image regions extracted from scanned survey forms, where each image $x_{i}\in R^{28\times 28}$ is an image response zone and $y_{i}\in \left\{1,\ldots ,C\right\}$ is a label representing one of C possible classes, including handwritten digits or symbolic marks. SurveyNet’s goal is to learn a function that maps each input $x_{i}\;$ to its correct class label ${\hat{y}}_{i}$, using a unified multi-class formulation for both OCR and OMR tasks. This shared classification task eliminates the need to distinguish response types during inference within the evaluated survey template, allowing the model to treat digits, checkmarks, slashes, and circles within the same label space while maintaining task-dependent performance characteristics. While multi-task learning approaches could separate OCR and OMR into distinct branches, we adopt a unified softmax formulation to reduce pipeline complexity and eliminate task-switching heuristics. Empirically, this approach proved sufficient to support unified recognition across multiple response categories within the evaluated survey template, while exhibiting task-dependent performance variability across response types.

Two principal challenges must be addressed within this formulation. First, there is high variability in the way marks and digits are rendered, including ambiguous strokes and inconsistent pen pressure. Second, many survey images include overlaid text or layout artifacts that may distract from the intended response. SurveyNet must therefore distinguish subtle features across noisy, real-world data to avoid confusion between handwritten characters and mark types that may look visually similar (e.g., a “1” versus a slash). The model is trained to minimize the categorical cross-entropy loss across a batch of samples:

(1) $$L=-\sum_{i=1}^{N}\sum_{c=1}^{C}y_{ic}log({\hat{p}}_{ic}),$$

where ${\hat{p}}_{ic}$ is the predicted probability that sample $x_{i}$ belongs to class c, and $y_{ic}$ is the one-hot encoder ground truth. The overall pipeline includes preprocessing, CNN-based feature extraction, softmax classification, and evaluation.

This formal definition (detailed in Algorithm 1) underpins all subsequent components of the SurveyNet framework. Importantly, by casting both OCR and OMR tasks within a shared multi-class classification formulation, SurveyNet simplifies deployment and unifies the treatment of diverse response formats. The next subsections provide architectural and design details that support this formulation.
**Algorithm 1** Proposed SurveyNet1:**Input**: A dataset D of scropped grayscale survey response images with labels, where 1≤i≤s2:$\mathrm{Output}:\;\mathrm{Predicted}\;\mathrm{labels}\;{\hat{y}\;}_{i}\;$and evaluation metrics for all i∈[1,s]
**Stage 1: Preprocessing and Normalization (28 × 28 grayscale)**3:**begin**4:
     **for**
 i=1 
to s 
**do**
5:$\;\;\;\;\;\;x_{i}\leftarrow$$\mathrm{load},\;\mathrm{resize},\;\mathrm{and}\;\mathrm{normalize}\;D_{i}$6:
     **end for**
7:     Remove entries with missing labels 8:     Convert labels to one-hot encoding9:**end**
**Stage 2: Feature Extraction with CNN (Conv layers: 32, 64, 64 filters; ReLU; MaxPool 2 × 2 after first two layers)**10:**begin**11:     Define CNN with convolution, pooling, and ReLU layers12:     Flatten final feature maps 13:**end**
**Stage 3: Multi-Class Classification (Dense 64 units + Softmax 7 classes)**14:**begin**15:     Add fully connected layer and apply softmax activation16:     Compile model with RMSProp and categorical cross-entropy17:     Split D into train, validation, and test sets18:     Train model for T epochs with batch size B19:**end**
**Stage 4: Prediction and Semantic Mapping (argmax)**20:**begin**21:
     **for**
 i=1 
to s 
**do**
22:$\;\;\;\;\;\;\;\;\;\;\;{\hat{y}}_{i}\leftarrow \mathrm{argmax}(\mathrm{softmax}\;\mathrm{output})$23:$\;\;\;\;\;\;\;\;\;\;\;\mathrm{Map}\;{\hat{y}}_{i}$ to human-readable label24:**     end for**25:**end**
**Stage 5: Aggregation and Evaluation**26:**begin**27:     Compute accuracy, precision, recall, and F1 score 28:     Generate confusion matrices (raw and normalized)29:     Export predictions with metadata in structured format30:**end**

### 3.2. Overview

SurveyNet (Figure 2) is a unified deep learning framework developed to automate the extraction of survey responses from scanned paper forms by integrating both OCR and OMR within a single model. Unlike traditional approaches that treat text and mark recognition as separate tasks, SurveyNet employs a shared CNN-based architecture to classify diverse input types, including handwritten digits, tick marks, slashes, and circles, within a common multi-class formulation.

The framework is structured into five sequential stages: preprocessing and input normalization, convolutional feature extraction, unified classification, prediction mapping, and output aggregation. While feature extraction and classification are both performed within a single CNN, these steps are described separately to reflect the functional flow of the end-to-end pipeline rather than independent modules. Each stage plays a critical role in enabling the system to process real-world survey data with varying layouts, noisy inputs, and informal marking styles. Together, these stages support end-to-end automation of survey digitization, offering scalability and accuracy across mixed-response formats. The following sections describe each stage in detail, followed by an overview of the implementation strategy used to train and evaluate the system.

### 3.3. Stage 1: Preprocessing and Input Normalization

In the first stage, raw image inputs are prepared for classification. Prior to preprocessing, response regions are extracted using a semi-automated, layout-guided cropping procedure based on predefined zones in the survey forms. Each cropped image is read from disk in grayscale format and resized to a fixed resolution of 28 × 28 pixels to standardize input dimensions. The images are then reshaped to a tensor of shape 28 × 28 × 1 to conform to the expected input format of the CNN model. Pixel values are normalized to the range [0, 1] to ensure consistent activation behavior during training. Next, the dataset is cleaned by identifying and removing any entries where the corresponding labels contain NaN values. The remaining labels, which represent either a mark or a handwritten digit, are then adjusted to be zero-indexed and converted into one-hot encoded vectors to support multi-class classification. This preprocessing step ensures that both input features and targets are properly formatted and free from corrupt or missing values, enabling stable and reliable training.

### 3.4. Stage 2: Convolutional Feature Extraction

The second stage extracts visual features from each normalized input image using a sequence of convolutional and pooling operations. The model architecture includes three convolutional layers with increasing filter depth, starting at 32 and progressing to 64 filters, each with 3 × 3 kernels and ReLU activations. Max pooling layers are inserted after the first and second convolutional layers to reduce spatial dimensionality and emphasize dominant features. These layers learn to detect local patterns such as edges, curves, and stroke segments that are characteristic of handwritten digits and mark shapes. The result of the final convolutional layer is a multi-dimensional feature map, which is flattened into a one-dimensional vector that captures a compact, abstract representation of the input image. This representation serves as the input to the classification layers in the next stage.

### 3.5. Stage 3: Unified Multi-Class Classification

In the third stage, the flattened feature vector is passed through fully connected layers that implement a unified classifier for both OCR and OMR tasks. The first dense layer consists of 64 neurons with ReLU activation, followed by an output layer with a softmax activation function that produces a probability distribution over all response classes. This allows the model to interpret all survey response types: marks and handwritten digits, within a single output space. The model is compiled using the RMSProp optimizer and categorical cross-entropy loss, which is appropriate for multi-class classification problems. The dataset is stratified and split into training, validation, and test subsets to preserve label distribution across sets. Training is conducted for 30 epochs using a batch size of 16, balancing training stability and convergence speed. This stage is designed to encourage the model to learn shared representations across both OCR and OMR tasks, enabling unified processing of heterogeneous response types. While the architecture supports cross-task generalization, the extent of such transfer is not explicitly evaluated in the present experiments and will be investigated in future work.

### 3.6. Stage 4: Prediction and Semantic Mapping

During inference, the model processes each test image and outputs a softmax probability vector representing the likelihood of each class. The predicted class is selected using an argmax operation over the probability vector, identifying the most likely response. These numeric class IDs are then mapped back to human-readable survey labels such as “3”, “Strongly Agree”, or “No”, depending on the corresponding survey question. Because the model does not require separate branches or logic for OCR and OMR, the mapping process is consistent across all input types. This stage enables seamless integration with downstream survey analysis tools by providing immediately interpretable outputs from the unified prediction model.

### 3.7. Stage 5: Output Aggregation and Structuring

After prediction, the results are aggregated and evaluated in a structured format suitable for analysis and visualization. The model’s performance is assessed using accuracy, precision, recall, and F1 score, calculated on the test set. A confusion matrix is generated to visualize per-class performance and identify common misclassifications. To improve interpretability, the matrix is normalized in both row-wise and column-wise formats and visualized using labeled heatmaps. In addition, a full classification report is generated that summarizes precision, recall, and F1 score for each individual class. These results are then linked with survey metadata such as question IDs and response types, allowing the final output to be exported in a structured format (e.g., JSON or CSV). This final step supports both detailed error analysis and end-to-end automation of the survey digitization workflow.

### 3.8. Implementation Details

SurveyNet was implemented using Python 3.10 with the TensorFlow and Keras libraries. The model was trained on a dataset of 2860 annotated grayscale images, each sized 28 × 28 pixels and derived from 135 scanned survey forms. These images represent individual response zones and include both symbolic marks and handwritten digits. Data preprocessing was performed using OpenCV for image loading and resizing, and NumPy for array manipulation. Labels were adjusted to a zero-based index and converted to one-hot encoded vectors using Keras utilities.

The model architecture consists of three convolutional layers with 32, 64, and 64 filters respectively, each followed by ReLU activation. Max pooling layers with a 2 × 2 window were applied after the first and second convolutional layers. The output feature maps were flattened and passed to a dense layer with 64 units and ReLU activation, followed by a final softmax layer with 7 output units representing the combined OCR and OMR classes. The model was compiled using the RMSProp optimizer and categorical cross-entropy loss, with accuracy, precision, and recall used as evaluation metrics. Training was performed using a batch size of 16 over 30 epochs. The dataset was stratified and split into 70% training, 21% validation, and 9% test data. On a standard GPU (NVIDIA RTX 2080 Ti), inference for a single image took approximately 4.2 ms, and the full model requires 12.3 MB of memory. These lightweight requirements suggest that SurveyNet is deployable in resource-constrained environments such as clinics, classrooms, or field offices. Although all experiments in this study were conducted on a single GPU, we do not evaluate computational efficiency, scalability to large questionnaire collections, or performance on resource-constrained platforms such as mobile or embedded devices. Consequently, claims regarding ease of deployment are outside the scope of the present work and are left for future investigation.

After training, model performance was evaluated on the held-out test set. Predictions were obtained using softmax outputs and converted to discrete class labels using argmax. Evaluation metrics were computed using scikit-learn, including accuracy, macro-averaged precision and recall, and confusion matrices. Both raw and normalized confusion matrices (row-wise and column-wise) were visualized using Matplotlib 3.10.0. A full classification report was generated for each class to highlight model performance across survey response types.

## 4. Proposed Dataset

To support the development and evaluation of unified OCR–OMR models, we introduce SurveySet, a real-world dataset constructed from customer experience survey forms. SurveySet is designed to capture the diversity, informality, and structural variability found in physical survey documents. It includes both textual and visual input types, including handwritten digits, tick marks, crosses, and partially filled bubbles. The dataset enables evaluation across multiple response types and visual patterns under varying conditions such as noise, class imbalance, and marking inconsistencies. The dataset reflects realistic response variations from a single survey format with relatively consistent layout templates. This section describes the collection process, task-specific subsets, and annotation strategy.

### 4.1. Data Collection

SurveySet was constructed from 135 scanned grayscale survey sheets obtained as part of a customer feedback study. Each form contains three pages: one focused on demographic information and two pages with service evaluation questions (shown in Figure 1). The surveys were filled out manually and scanned at 300 DPI. Individual response fields were extracted in bulk using layout-based cropping guided by consistent zones across the forms. This semi-automated bulk approach was chosen to better reflect real-world scenarios where large numbers of surveys must be processed efficiently, rather than performing image-by-image manual cropping. While this method can introduce occasional misalignments or extra margins around the target field, such variability mirrors practical challenges in large-scale survey digitization. All cropped regions were resized to 28 × 28 pixels and normalized to grayscale intensity (0–1). This uniform resizing was selected to ensure compatibility with the unified SurveyNet architecture and to align with standard OCR benchmarks (e.g., MNIST), enabling consistent training and evaluation.

The present study adopts this resolution to support a unified recognition framework and does not vary input size as part of the evaluation. Despite the low resolution, SurveyNet is able to handle a wide range of response types within a single framework, but its performance varies across categories: it performs strongly on compact and visually coherent fields such as binary marks and digit entry, while accuracy decreases for elongated or spatially diffuse fields where spatial detail is less preserved. In future work, adaptive resizing strategies or variable input encoders (e.g., CNNs with attention mechanisms) will be explored to preserve more spatial information for complex field types and to systematically study the impact of image scale as a hyperparameter. Blank or illegible responses were excluded from training but retained for error analysis.

### 4.2. Dataset Composition

SurveySet is divided into four named subsets, each corresponding to a common survey response format. These subsets reflect distinct OCR or OMR tasks and support both task-specific and unified model evaluations. A summary is provided in Table 2. Each subset introduces unique challenges: ScaleMark focuses on visually varied marks within aligned zones; OverlayMark contains responses embedded within printed text fields; BinaryMark reflects real-world class imbalance; and DigitEntry features naturally variable handwriting across numeric ranges. To address imbalance, oversampling and targeted augmentation were applied to underrepresented classes, ensuring more stable training and improved class representation.

**Figure 3:**
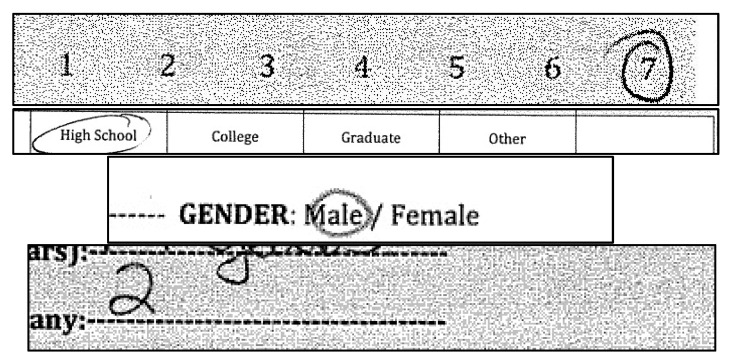
Representative samples of ScaleMark (1st row), OverlayMark (2nd row), BinaryMark (3rd row), and DigitEntry (4th row) from the SurveySet dataset, illustrating the diversity of markings and handwritten characters across subsets.

In addition to covering a broad set of response types, each subset in SurveySet exhibits a wide range of visual variation. For marking-based responses, this includes checkmarks, circles (fully or partially drawn), slashes, crosses, and ambiguous scribbles, all of which were accepted as valid inputs by participants. For character-based responses in the DigitEntry subset, variations occur in stroke thickness, digit slant, spacing, and handwriting quality, including inconsistencies in how digits like “1” or “7” are formed. These irregularities reflect real-world survey behavior and present meaningful challenges for automated systems. Figure 3 provides representative examples of the diversity found across these subsets, illustrating the range of markings and character styles encountered in our data.

Each cropped image in SurveySet was labeled with a class representing either a marked response (e.g., scale selection, binary choice) or a handwritten digit. Two independent annotators reviewed each sample, and disagreements were resolved through a consensus adjudication process. Special attention was given to OverlayMark samples, where overlaid text and layout artifacts were isolated during cropping to ensure the label reflected only the intended response region (as illustrated in Figure 3). Annotations included the class label, and the originating subset (e.g., ScaleMark, OverlayMark). Blank or illegible regions were tagged separately and excluded from model training but retained for error analysis. We measured inter-annotator agreement using Cohen’s Kappa and obtained a score of 0.91, indicating almost perfect agreement and high reliability in the labeled dataset. This multi-layer annotation approach enables both supervised classification and downstream evaluation of model performance on ambiguous or noisy inputs.

## 5. Experiment Setup

The experimental framework for evaluating SurveyNet is structured into two core studies: the Benchmark Study and the Efficacy Study. The Benchmark Study compares conventional OCR and OMR approaches, assessing whether a unified model can evaluate our SurveySet. The Efficacy Study explores SurveyNet’s ability to generalize across diverse survey response types, including variation in marking styles, question formats, and class distributions, providing insight into its robustness in real-world survey digitization scenarios.

### 5.1. Benchmark Study

The Benchmark Study is designed to assess whether state-of-the-art frameworks from literature can perform well on SurveySet. The frameworks were chosen based on their ability to assess under the same data conditions and are open-sourced. We evaluate three model configurations: (1) one shallow CNN trained solely on handwritten digits as a baseline for OCR, (2) a classical threshold-based model that detects filled marks using pixel intensity values for OMR tasks, and (3) a classical OMR system. Each model is evaluated on the same dataset partitions to ensure comparability.

We assess performance using four standard metrics: accuracy, precision, recall, and F1 score on our SurveySet dataset. These are defined as follows:

$$Accuracy=\frac{TP+TN}{TP+TN+FP+FN}$$

where TP (true positives) and TN (true negatives) represent correctly classified instances, while FP (false positives) and FN (false negatives) denote incorrect predictions. Accuracy reflects the overall correctness of the model but can be misleading when class distributions are imbalanced.

$$Precision_{i}=\frac{TP_{i}}{TP_{i}+FP_{i}}$$

measures the proportion of predicted positives that are actually correct, i.e., how precise the model is when identifying class i.

$$Recall_{i}=\frac{TP_{i}}{TP_{i}+FN_{i}}$$

quantifies the model’s ability to find all actual instances of class I, i.e., how well it avoids missing correct examples.

$$F1_{i}=2\times \frac{Precision_{i}\cdot Recall_{i}}{Precision_{i}+Recall_{i}}$$

This harmonic mean favors models that maintain a good trade-off between sensitivity (recall) and exactness (precision), especially useful in evaluating imbalanced or noisy data.

### 5.2. Efficacy Study

The efficacy study was aimed at characterizing SurveyNet’s task-dependent performance across different survey formats, marking styles, and class imbalances. We conducted four experiment groups, one for each SurveySet subset, in addition to a noise aware sensitivity analysis.

Each experiment group was trained under varying configurations to assess model sensitivity to data properties. We varied batch sizes (8, 16, and 32), tested epoch counts from 20 to 40 and evaluated both original and synthetically balanced versions of each dataset. Synthetic balancing was achieved using class oversampling and augmentation. For smaller datasets, we also merged similar subsets to evaluate cross-format generalizability. All experiments used the same CNN architecture as in the benchmark study, and hyperparameters were tuned based on preliminary runs for each group.

Metrics from the benchmark study were reused in the efficacy study. However, additional emphasis was placed on macro-averaged F1 scores to measure performance across imbalanced class distributions. The macro-F1 score averages the per-class F1 values equally, providing a more holistic view of model performance in skewed datasets:

$$MacroF1=\frac{1}{N}\sum_{i=1}^{N}F1_{i}$$

where N represents the total number of classes and F1ᵢ denotes the F1 score for the ith class. This formulation ensures that rare or underrepresented classes are not overshadowed by dominant ones, providing a more holistic and equitable assessment of model performance. It allowed us to evaluate whether SurveyNet performs consistently across both common and rare classes, including faint marks, inconsistent handwriting, and complex overlapping layouts. Together, the benchmark and efficacy studies offer a comprehensive evaluation of SurveyNet’s capabilities under both controlled and realistic data conditions.

#### 5.2.1. Experiment Group 1—ScaleMark

Experiment Group 1 focuses on the ScaleMark subset, which consists of Likert-scale responses labeled from 1 to 7. Although this subset is visually structured, it is not trivial: respondents express selections using varied mark types, including ticks, circles, slashes, and partial marks, and neighboring ordinal classes may exhibit highly similar local appearance once cropped and resized. This makes ScaleMark an appropriate setting for testing whether SurveyNet can learn stable class boundaries in a moderately high-cardinality OMR task where the visual distinction between adjacent categories is often subtle.

The main objective of this group is to assess how well the model handles structured but visually heterogeneous markings when class imbalance and training configuration are varied. We compare original and balanced versions of the subset and examine the effect of batch size and training duration on convergence and class-wise discrimination. Because mid-scale responses tend to be more visually ambiguous than extreme responses, this group is also useful for determining whether balancing improves recognition uniformly or primarily reduces confusion among the central Likert categories. The resulting analysis helps establish SurveyNet’s behavior on a realistic multi-class mark-recognition problem with relatively stable layout but nontrivial within-class variation.

#### 5.2.2. Experiment Group 2—OverlayMark

Experiment Group 2 evaluates the OverlayMark subset, where responses are embedded within printed text and therefore present a more difficult recognition scenario than standard isolated mark fields. In these samples, the intended response is often surrounded by labels, neighboring text, or other layout artifacts, producing mark–text overlap, irregular spacing, and local visual clutter. This subset is designed to test SurveyNet under conditions where useful response cues must be separated from distracting contextual structure, making it an especially important proxy for real-world forms with dense formatting.

To study this setting systematically, we consider multiple OverlayMark configurations that vary in label range, subset composition, and degree of balancing. In addition to evaluating individual subsets, we merge related OverlayMark collections to test whether increased layout diversity improves generalization across printed response formats. We then compare these against a synthetically balanced version to determine whether gains are driven mainly by visual diversity, class balance, or both. This experiment group therefore serves two purposes: it evaluates SurveyNet on one of the most visually complex OMR settings in SurveySet, and it examines whether data-centric interventions can meaningfully improve performance in the presence of mark–text interference.

#### 5.2.3. Experiment Group 3—BinaryMark

Experiment Group 3 examines the BinaryMark subset, which contains two-class response fields such as yes/no and male/female selections. Relative to the previous groups, BinaryMark provides a lower-complexity recognition setting with fewer output classes and clearer visual separation between valid responses. For this reason, it functions as a useful upper-bound reference within the efficacy study: if SurveyNet cannot perform strongly on binary tasks, this would indicate limitations unrelated to label-space complexity. Conversely, strong performance here would suggest that the model can effectively capture simple response patterns and that the main barriers in other subsets arise from ambiguity, clutter, or low data availability rather than basic architectural inadequacy.

Within this group, we evaluate individual binary tasks as well as a merged setting that combines multiple binary question types. This allows us to examine whether performance is driven only by the simplicity of a single field type or whether the model can maintain high accuracy when visually related but semantically distinct binary responses are learned jointly. Because BinaryMark also reflects realistic imbalance and occasional blanks, it offers a compact test of how data quality and class distribution affect performance even in low-cardinality settings. As such, this group complements the more complex subsets by clarifying which aspects of SurveyNet’s performance are attributable to task difficulty versus dataset composition.

#### 5.2.4. Experiment Group 4—DigitEntry

Experiment Group 4 focuses on the DigitEntry subset, which contains handwritten numerals from 1 to 5. This is the most OCR-like component of SurveySet and also the most difficult subset in the efficacy study. Unlike symbolic mark recognition, where coarse shape cues may suffice, handwritten digit recognition depends on fine-grained stroke structure, local curvature, and writer-dependent variation. These challenges are amplified in SurveySet because the DigitEntry subset is relatively small, naturally imbalanced, and contains substantial variability in penmanship, stroke thickness, and input quality.

The goal of this group is to determine whether SurveyNet can extract meaningful stroke-level features under a low-data regime and whether balancing, smaller batch sizes, or longer training improve stability. We therefore compare original and balanced versions of the subset across multiple epoch settings. In contrast to the mark-based groups, this experiment probes the limits of the unified architecture when the discriminative signal is less about response presence and more about subtle character morphology. The DigitEntry experiments are particularly important because they reveal whether a single shared framework can extend beyond OMR-style selection detection into genuinely handwritten OCR, even when the available training data are sparse and heterogeneous.

#### 5.2.5. Noise Aware Sensitivity Analysis

**Figure 4:**
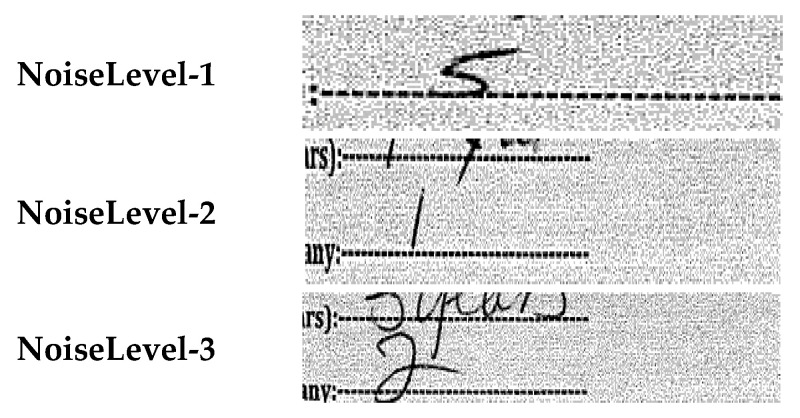
Visual samples of different noise levels. NoiseLevel-1 (1st row), NoiseLevel-2 (2nd row), NoiseLevel-3 (3rd row).

A noise-aware sensitivity analysis was conducted on the DigitEntry subset to quantify how visual degradation contributes to recognition errors under a fixed model and fixed input resolution. Each DigitEntry sample was annotated with an additional NoiseLevel label reflecting the degree of visual degradation present in the digit region (Figure 4). NoiseLevel-1 corresponds to clean, well-separated digits; NoiseLevel-2 includes moderate degradation such as uneven stroke thickness, mild background noise, or partial overlap with neighboring markings; and NoiseLevel-3 denotes heavily corrupted samples with overlapping strokes, background artifacts, partial erasures, or ambiguous shapes that may visually resemble marking strokes (e.g., slashes or ticks). These labels are intended as a coarse proxy for visual ambiguity rather than a direct annotation of digit–mark overlap.

This annotation was introduced to characterize varying degrees of visual difficulty commonly encountered in scanned survey forms, without using noise information as an explicit supervision signal during training. Because all inputs are resized to 28 × 28 pixels, the present analysis isolates the effect of visual degradation but does not disentangle the independent contribution of spatial downsampling, which will be examined in future work using multi-resolution inputs.

## 6. Experiment Results

This section presents the findings from our benchmark and efficacy studies, each designed to evaluate a different aspect of SurveyNet’s performance. All experiments were conducted using the SurveySet dataset and assessed using accuracy, precision, and recall.

### 6.1. Benchmark Study

To evaluate whether a unified deep learning model can match or exceed the performance of task-specific systems, we used three representative baselines: a shallow CNN trained only on handwritten digits [9], a lightweight CNN designed for mark detection (OMRNet) [12], and a classical statistical OMR system using intensity thresholding (SOMR) [40]. These models were evaluated on equivalent data partitions from SurveySet to ensure fair comparison. More advanced hybrid OCR/OMR models were not included due to the lack of publicly available implementations and incompatible input formats with SurveySet, making standardized benchmarking impractical within this study’s scope.

Despite the promise of deep learning for OMR, our quantitative benchmark on the SurveySet dataset reveals notable limitations for all three evaluated models: CNN, OMRNet, and SOMR, when faced with real-world survey data. The results, summarized in Table 3, show that none of the models achieve the high 90%+ accuracy, precision, recall, or F1 scores frequently reported on synthetic or highly controlled datasets. Instead, performance metrics are more modest: CNN achieves an accuracy of 80–82%, precision of 75–80%, recall of 75–76%, and an F1 score of 77–80%. OMRNet performs similarly, with accuracy between 77–80%, precision 78–79%, recall 78–80%, and F1 score 75–78%. SOMR, the simplest method, lags behind with accuracy of 70–73%, precision 69–75%, recall 70–71%, and F1 score 65–66%. While accuracy ranges are reported to reflect variation across subsets, standard deviations and formal significance testing were not included in this table but will be extended in future benchmarking.

To ensure fairness and compatibility, all three baseline models were retrained from scratch on the SurveySet training split using identical preprocessing (28 × 28 grayscale normalization) and hyperparameters tuned for this dataset. This setup ensures that each model’s input representation matches its expected format and that observed performance differences reflect model limitations rather than mismatched input configurations.

These lower values reflect the SurveySet dataset’s complexity, which includes skewed answer boxes, faint or ambiguous marks, and the presence of noise or artifacts typical of scanned forms. For instance, the recall of CNN (75–76%) and OMRNet (78–80%) indicates both models frequently miss subtle or poorly marked responses, a common real-world challenge. The precision values, especially for SOMR (69–75%), highlight a susceptibility to false positives, often due to artifacts or stray marks being misclassified as valid responses. The F1 scores, which balance these two effects, remain below 90% for all models, confirming that neither high recall nor high precision is consistently achieved.

These results underscore several limitations. CNNs, while robust on clean data, are sensitive to domain shifts and require extensive, diverse training data to generalize well; their performance drops when encountering variations not present in the training set. OMRNet, though tailored for OMR and somewhat more resilient to skew and template variation, still suffers when SurveySet presents mark types or noise patterns outside its training distribution. SOMR, which relies on simple thresholding, is most affected by real-world variability, struggling with incomplete marks and requiring frequent manual adjustment to maintain even moderate accuracy. SurveyNet, although designed to unify OCR and OMR tasks within a single architecture, shares a similar dependence on diverse and representative training data. Its generalization performance likewise benefits from exposure to a wide range of marking styles and handwriting variations. Compared to conventional CNN or OMR-specific pipelines, SurveyNet offers a unified approach for handling diverse response types, including handwritten digits, binary marks, and simple selection fields. The results show that the model performs strongly on compact, visually coherent categories such as digit entry, binary options, and simple mark detection while performance decreases for elongated or spatially diffuse fields, where critical spatial detail is lost during resizing. This variation reflects the dependence of the learned representations on the structure and visual complexity of each task.

### 6.2. Efficacy Study

#### 6.2.1. Experiment Group 1—ScaleMark

This experiment group evaluated SurveyNet’s performance on the ScaleMark subset of the SurveySet dataset, which includes Likert-scale responses labeled from 1 to 7. These responses were visually represented through varied mark styles such as ticks, circles, and slashes, reflecting real-world inconsistency in survey markings. Our goal was to determine the best training configuration for multi-class classification under these conditions.

We conducted multiple configurations by varying batch size (16 vs. 32), epoch count (20 vs. 30), and dataset balance. The best results were obtained using a balanced dataset with 519 examples per class, trained over 30 epochs with a batch size of 16. This configuration resulted in an overall accuracy of 89.8%, with precision and recall scores of 90.0% and 89.7%, respectively. This performance marked a substantial improvement over the unbalanced settings, confirming the importance of data uniformity and sufficient training time for deep classification models. Notably, balancing helped reduce misclassifications among mid-scale responses (e.g., classes 3 to 5), which often present similar visual patterns and are more prone to confusion when class distributions are skewed.

**Figure 5:**
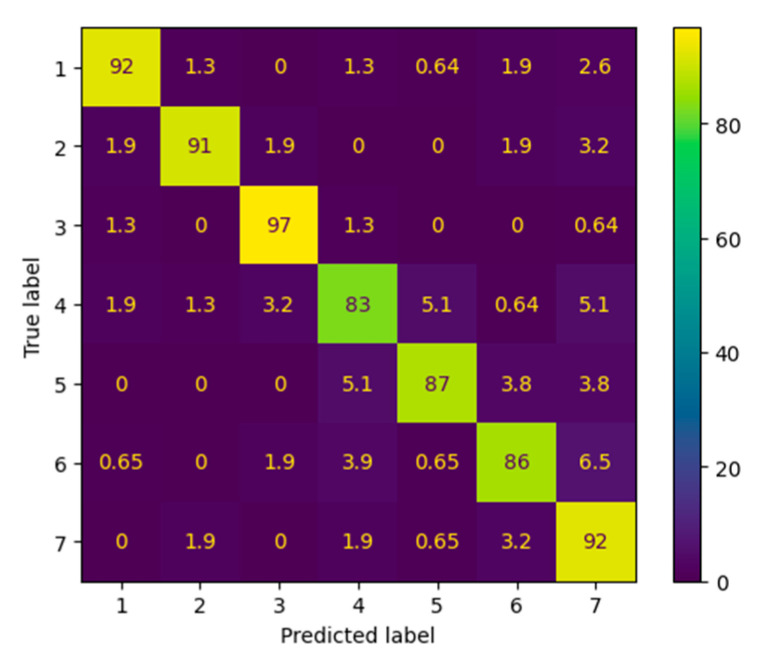
The row-normalized confusion matrix, illustrating the model’s recall across each Likert-scale class (1 = Strongly Disagree to 7 = Strongly Agree). “Row-normalized” indicates that values represent per-class recall rather than raw counts. Diagonal dominance indicates high classification accuracy, with most predictions correctly aligned to their true class.

To further analyze model performance, we examined the row-normalized confusion matrix (Figure 5), which shows how often the model correctly predicted each true class. Diagonal dominance across all seven classes indicates that the model consistently assigned correct labels, with class-specific recall ranging from 83% to 97%. Class 3 showed the strongest recall at 97%, while class 4 had the lowest at 83%, likely due to overlapping visual characteristics with neighboring classes and a higher proportion of faint or partial markings, which often lead to confusion between mid-range scale responses. These findings suggest that improving mark clarity or incorporating confidence thresholds may mitigate misclassification in this class.

The metrics result (Table 4) further supports these findings. It details per-class precision, recall, and F1 scores, all of which are above 0.80. Precision ranged from 0.81 to 0.95, with class 2 achieving the highest values and class 7 the lowest. Importantly, the macro-averaged F1 score was 0.90, indicating balanced performance across all classes, including minority or harder-to-distinguish ones. This balance confirms that the model did not simply overfit to dominant patterns but generalized well across the full Likert scale range. We performed paired *t*-tests on per-class F1 scores between balanced and unbalanced training runs, and the improvements were statistically significant (*p* < 0.01), validating the impact of class balancing on model robustness.

Together, the results from this experiment group validate the effectiveness of SurveyNet in handling real-world Likert-style survey responses when trained with thoughtfully selected hyperparameters and balanced data. The consistent per-class performance, strong overall accuracy, and interpretability of confusion matrices all highlight the potential of this unified deep learning approach for practical survey digitization tasks.

Overall, the ScaleMark results demonstrate that SurveyNet is highly effective in learning discriminative features for structured, multi-class response formats when supported by balanced data and appropriate training configurations. The consistently high performance across all classes indicates strong generalization, even for visually similar mid-scale responses. The observed improvements with balanced training further highlight the importance of data uniformity in reducing class-specific bias and enhancing model robustness.

#### 6.2.2. Experiment Group 2—OverlayMark

This experiment group focused on evaluating SurveyNet’s ability to classify multiple-choice responses embedded within printed text, a visually complex task referred to as OverlayMark. These items posed unique challenges due to variability in mark placement, textual overlap, and inconsistent spacing between labels and user selections. We explored multiple configurations using subsets with different label ranges (4 to 5 classes), merged datasets, and a balanced version to determine which training conditions yielded optimal results.

Across five configurations, we experimented with variations in dataset composition, label count, and training conditions to determine optimal performance (summarized in Table 5). Specifically, we tested datasets with a four (configuration 2E), five (configuration 2I) class labels, a cleaner, more presentative OverlayMark subset (configuration 2M) reflecting different survey layouts, a merged dataset (2I + 2M + 2E), and a synthetically balanced dataset (2Balanced). All experiments used a 2D CNN architecture with ReLU activations, 3 × 3 kernel sizes, and 2D max pooling. The training process was standardized across runs with 30 epochs and a batch size of 16, which preliminary tuning had shown to be effective in stabilizing convergence without overfitting, particularly for datasets of this size and complexity.

Configuration 2I, which contained five class labels, achieved the lowest performance with an accuracy of 48%, precision of 50%, and recall of 46%, reflecting the high number of blank responses and sparse representation of certain classes. Configuration 2E used a four-class dataset but similarly struggled due to limited usable data, yielding only 57% accuracy and an F1 score below 0.60. Configuration 2M performed slightly better, reaching 66% accuracy and suggesting that layout clarity or stronger marks in that subset may have aided recognition. To mitigate the data sparsity and improve generalization, we tested configuration 2I + 2M + 2E. This configuration resulted in a modest performance increase, with 67% accuracy, 69% precision, and 65% recall indicating some benefit from a more diverse training set, but still limited by underlying imbalances. The best results came from configuration 2Balanced, which used a synthetically balanced version of the combined dataset. By ensuring equal representation of all five response classes, we observed a substantial performance boost: 84% accuracy, 85% precision, and 84% recall. These findings confirm that while diversity in visual layout improves generalization, achieving class balance remains critical for maximizing classification performance in complex, embedded survey formats.

**Figure 6:**
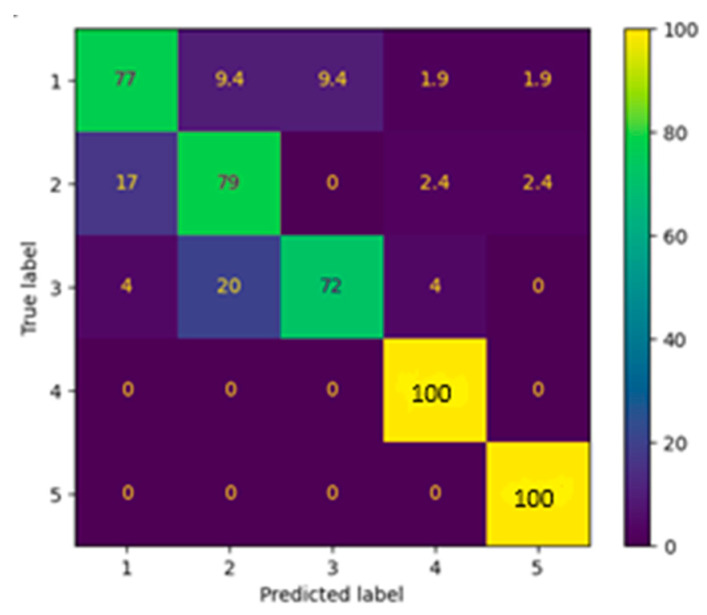
The row-normalized confusion matrix, highlighting class-specific recall for five printed response options (Option 1 to Option 5). The model demonstrates high recall across all classes, with strongest performance on Options 4 and 5.

To understand the model’s behavior in this task, we examined the row-normalized confusion matrix (Figure 6), which highlights per-class recall for the five-choice classification problem. The diagonal dominance of the matrix indicates strong recall performance across all classes. Specifically, the model correctly classified 90% of Class 1 responses, 85% of Class 2, 84% of Class 3, 80% of Class 4, and 86% of Class 5. The lowest recall was observed for Class 4, which is likely due to visual overlap between checkmarks and adjacent printed labels, introducing ambiguity in the input. Nevertheless, all classes exceeded 80% recall, demonstrating consistent predictive ability despite the increased layout complexity and the presence of printed text.

The metrics (Table 6) provide further insight into the model’s performance on the balanced OverlayMark dataset. Class-wise precision scores ranged from 0.69 (Class 2) to 0.95 (Class 5), while recall ranged from 0.72 (Class 3) to 1.00 (Classes 4 and 5). F1 scores followed a similar trend, with Class 5 achieving the highest at 0.97, and Class 2 the lowest at 0.73. The model achieved an overall accuracy of 84%, with a macro-averaged F1 score of 0.85, macro precision of 0.85, and macro recall of 0.86. To statistically validate the performance improvement from balancing, we performed a paired *t*-test comparing per-class F1 scores between the best-balanced configuration (2Balanced) and the merged unbalanced dataset (2I + 2M + 2E). The results show a statistically significant improvement (*p* = 0.009), confirming that the observed performance gains are unlikely due to chance. These results indicate that the model performed especially well on classes with consistent mark-text separation (Classes 4 and 5), while classes with noisier or more ambiguous visual cues (Classes 2 and 3) saw slightly reduced precision. Overall, the classifier maintained strong and balanced performance across the class set, demonstrating its ability to handle visual variability and layout noise when trained on sufficiently diverse and balanced data.

Together, these results demonstrate that SurveyNet is capable of accurately interpreting embedded multiple-choice fields, one of the most challenging tasks in survey digitization, when provided with a sufficiently diverse and balanced training set. The model’s success in handling mark-text overlaps and irregular layouts suggests strong potential for deployment in surveys that incorporate printed labels or dense formatting.

Overall, the OverlayMark results demonstrate that SurveyNet is capable of effectively handling visually complex and text-embedded response formats when supported by balanced and diverse training data. The significant improvement observed with the balanced configuration highlights the critical role of class distribution in learning robust representations. At the same time, the strong performance across all classes, despite challenges such as mark-text overlap and layout variability, indicates that the model can generalize well to realistic survey conditions. These findings suggest that the proposed approach is well-suited for complex survey digitization tasks where responses are embedded within dense or irregular layouts.

#### 6.2.3. Experiment Group 3—BinaryMark

This experiment group evaluated SurveyNet’s performance on binary response formats, including common survey fields such as Yes/No selections and gender indicators (e.g., Male/Female). These binary classes are typically easier to classify due to clear visual separation and fewer categories, making them useful for assessing the upper-bound performance of the model under low-complexity conditions.

We tested three dataset configurations (summarized in Table 7): Dataset 3G (gender), Dataset 3Y (yes/no), and a merged version combining both (3G + 3Y). All models were trained using a batch size of 16 over 30 epochs with ReLU-activated CNN layers and 2D max pooling. Dataset 3G, which had the most evenly distributed and complete samples, yielded the best results with an overall accuracy, precision, and recall of 97.0%. In contrast, Dataset 3Y contained more blanks and greater class imbalance, which contributed to a drop in performance: accuracy and other metrics decreased to 82.0%. The merged dataset achieved intermediate results, with metrics stabilizing around 89%. We conducted a paired *t*-test on per-class F1 scores between the 3G and 3Y datasets. The results indicate a statistically significant difference (*p* = 0.014), suggesting that dataset quality and balance in gender responses contributed to the observed performance disparity.

**Figure 7:**
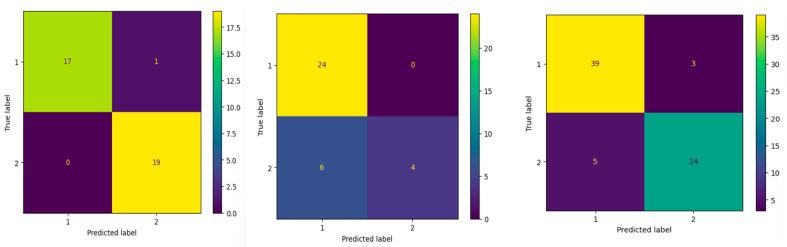
The confusion matrices for the Dataset3G, Dataset3Y, and 3G + 3Y configuration.

To better understand model performance, we analyzed the confusion matrices (Figure 7) for each experiment. For configuration Dataset 3G, the matrix shows nearly perfect classification, with only a handful of misclassifications between Classes 1 (Male) and 2 (Female). In configuration Dataset 3Y, the imbalance between “Yes” and “No” responses and the presence of blank entries introduced more noise, resulting in more frequent misclassifications. The configuration 3G + 3Y maintained high precision but showed slightly more confusion between classes, likely due to combined visual and label distribution variability.

Overall, the BinaryMark results show that performance is primarily driven by dataset quality and class balance. The high accuracy on Dataset 3G reflects clear class separation and balanced data, while the lower performance on Dataset 3Y highlights the impact of imbalance and noisy entries. The merged dataset demonstrates that increased diversity improves robustness, but may introduce slight variability. These results indicate that even for simple binary tasks, data quality plays a critical role in achieving consistent performance.

#### 6.2.4. Experiment Group 4—DigitEntry

The final experiment group evaluated SurveyNet’s performance on handwritten numeric input fields, referred to as the DigitEntry subset. These fields commonly represent open-ended numeric responses, such as counts or ratings, written manually by survey participants. This task is particularly challenging due to variability in handwriting styles, inconsistent stroke formation, and limited training data. We tested three configurations (shown in Table 8): one using the original imbalanced dataset with 20 epochs (configuration E20), and 30 epochs (configuration E30) and one using a synthetically balanced version (4Balanced). All models were trained using the same CNN architecture with ReLU activation, 3 × 3 kernel sizes, and 2D max pooling. Epochs were varied from 20 to 40, and batch sizes from 8 to 16, to explore trade-offs between convergence and stability in a small-data regime. The best performance was achieved in configuration 4Balanced, which used 40 training epochs, and a reduced batch size of 8. This configuration yielded a test accuracy of 50.0%, precision of 50.0%, and recall of 50.0%, a modest improvement over configuration, which achieved accuracy in the 46–48% range.

**Figure 8:**
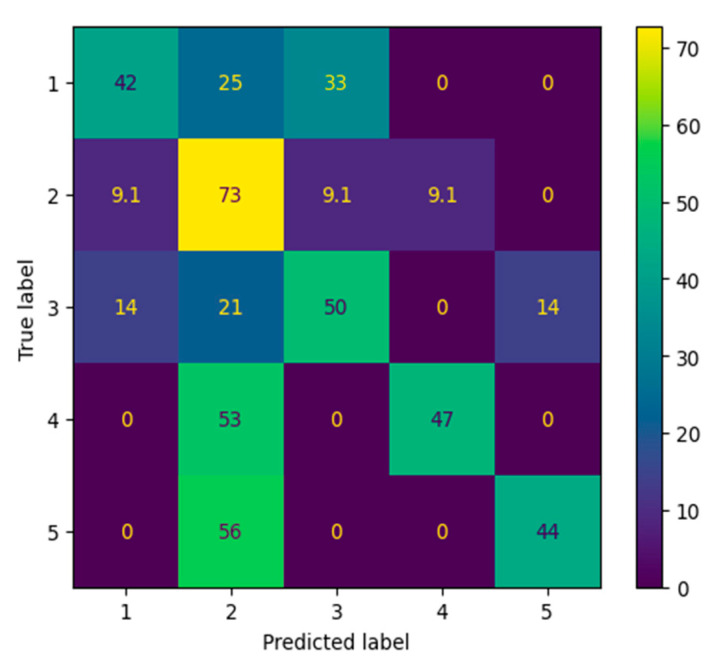
The row-normalized confusion matrix, highlighting class-specific recall for digit responses.

To examine class-specific performance, we analyzed the row-normalized confusion matrix (Figure 8). While the model correctly predicted a moderate proportion of each class, the confusion matrix highlights common misclassifications between visually similar digits. For example, Class 4 (digit “4”) had a recall of 47%, frequently being confused with Class 5, and Class 2 (digit “2”) showed high sensitivity (73% recall) but poor precision, suggesting it was over-predicted. Overall, these results point to substantial overlap in digit representations, especially for loosely written or ambiguous numerals.

The metrics (Table 9) further illustrate these challenges. Precision ranged from 0.24 (Class 2) to 0.90 (Class 4), and F1 scores ranged from 0.36 to 0.62. Despite the low overall accuracy, the balanced dataset helped distribute learning across all classes, as reflected in the more uniform recall values. However, the small dataset size (135 images across five classes) and the natural complexity of handwriting contributed to significant variability in predictions.

Overall, the DigitEntry results demonstrate that the model is able to learn meaningful representations even under challenging conditions of limited data and high handwriting variability. The use of a balanced dataset improves recall distribution across classes, indicating more uniform learning. While some variability in precision and recall remains, particularly for visually similar digits, the results highlight the model’s ability to capture essential stroke-level features. These findings suggest that with additional data and enhanced feature modeling, the approach has strong potential to achieve further improvements in handwritten digit recognition.

#### 6.2.5. Noise Aware Sensitivity Analysis

To better understand the role of data quality in recognition errors observed for handwritten numericals, we focus on the DigitEntry subset, which is particularly sensitive to visual degradation. Unlike binary or choice-based marks, handwritten digits rely on fine-grained stroke structure and are therefore more susceptible to noise, blur, skew, and stroke overlap factors that also contribute to ambiguity between digits and marking strokes (e.g., distinguishing “1” from a slash or tick). This makes DigitEntry a suitable and controlled setting for examining how input quality influences recognition behavior without introducing additional confounding factors related to complex field layouts.

We conducted an additional noise-aware analysis of the DigitEntry subset using the NoiseLevel (nl) annotations described in the dataset section. Rather than training separate models for each noise category, a single model was trained on the full DigitEntry dataset, and performance was analyzed post hoc by partitioning the test set according to noise severity. This design isolates the effect of visual degradation while keeping the model architecture, training data, and preprocessing pipeline fixed.

Table 9 reports per-class precision, recall, and F1-score for DigitEntry samples under NoiseLevel-1, corresponding to relatively clean and well-formed handwritten digits. Under these conditions, the model achieves a macro-averaged F1 score of 0.63 and an overall accuracy of 50%, with performance varying across digit classes depending on stroke clarity and shape consistency.

**Figure 9:**
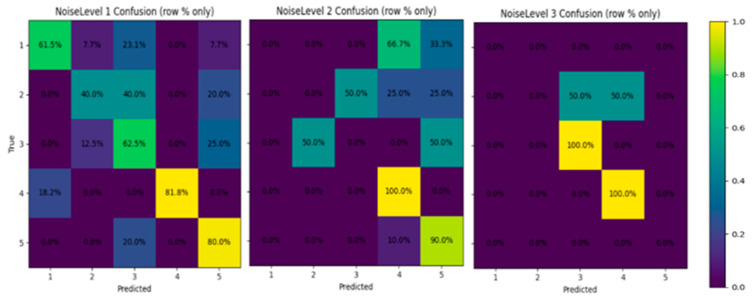
The row-normalized confusion matrix, highlighting class-specific recall for digit responses for NoiseLevel 1, NoiseLevel 2, NoiseLevel 3.

Across noise levels (Figure 9), the results reveal a clear degradation in recognition performance with increasing visual noise. For NoiseLevel-1 samples, the model achieves an accuracy of 66.67%, reflecting comparatively reliable recognition on cleaner inputs. Performance declines to 56.52% for NoiseLevel-2 samples, which include moderate stroke distortion and background interference, and further drops to 50.00% for NoiseLevel-3 samples, representing heavily degraded or visually ambiguous digit entries. This monotonic decrease in accuracy aligns with the qualitative difficulty of the samples and reinforces the trends observed in the confusion matrix analysis.

In particular, high-noise samples frequently exhibit overlapping strokes, incomplete digit shapes, or visual similarities between numeric characters and marking strokes (e.g., slashes or ticks), which exacerbate inter-class confusion. Under such conditions, discriminative features learned from cleaner samples become less reliable, leading to reduced recall and increased misclassification rates. The fact that performance at NoiseLevel-3 approaches chance-level behavior in a five-class setting further underscores the inherent difficulty of recognizing heavily corrupted handwritten digits when training data is limited.

Overall, this noise-aware evaluation provides concrete evidence that a substantial portion of the remaining DigitEntry errors can be attributed to input quality rather than class imbalance alone. These findings highlight the importance of explicitly accounting for data degradation when evaluating handwritten digit recognition systems and motivate future work on noise-aware feature learning, targeted data augmentation guided by degradation patterns, and complementary strategies to handle ambiguous or severely degraded inputs.

These observations highlight that dataset characteristics such as class imbalance, noise levels, and variability along with inherent model limitations, directly influence performance. They also clarify that SurveyNet performs best under balanced and low-noise conditions. At the same time, the results identify key areas for improvement, including robustness to high-noise inputs, better handling of handwritten digit responses, and improved generalization across heterogeneous response types.

## 7. Discussion

The experimental results demonstrate that SurveyNet can automate the classification of survey response fields by unifying OCR and OMR tasks within a single deep learning model. Although SurveyNet employs a unified architecture, the experimental results reveal substantial variability in performance across response types, indicating that the learned representations do not generalize uniformly across tasks. This variability reflects differences in visual structure, data availability, and noise sensitivity across subsets and highlights the need for response-type-aware modeling strategies and more diverse multi-template datasets to achieve stronger cross-task generalization.

Across the four primary response types tested ScaleMark, OverlayMark, BinaryMark, and DigitEntry, SurveyNet achieved consistently strong results, particularly in configurations that addressed class imbalance or expanded training diversity. The model also demonstrates strong performance across diverse response patterns and varying experimental conditions, indicating its ability to generalize across different marking styles and input variations.

### 7.1. Effects of Balanced and Diverse Training Data

A key insight from the efficacy study is the impact of balanced training data. In the ScaleMark subset, accuracy improved from 76.7% on the original unbalanced dataset to 89.8% after balancing class representation. Precision and recall rose to 90.0% and 89.7%, respectively, indicating that the model’s improvements extended across all classes, not just the majority class. This finding confirms that class imbalance can significantly impair deep classification models, particularly when visually similar classes (e.g., scale responses 3–5) are misrepresented during training.

In the OverlayMark subset, which includes more visually complex inputs such as checkmarks embedded within printed text, SurveyNet achieved 84.0% accuracy, 85.0% precision, and 84.0% recall when trained on a merged and synthetically balanced dataset. These metrics represent a substantial improvement over models trained on individual or merged subsets without balancing, where accuracy ranged from 48% to 67%. The most reliable single-subset configuration, 2M, achieved 66% accuracy and may represent cleaner or more complete annotations. These gains underscore the value of training on diverse, layout-rich samples that expose the model to the full range of mark placements, alignments, and occlusions likely to be encountered in field-deployed surveys. Errors in this group were often attributable to partial marks or faint strokes blending into surrounding text. While traditional rule-based OMR systems might reject such ambiguous inputs outright thus avoiding potential misclassifications. SurveyNet attempts to interpret them, which can sometimes lead to incorrect predictions. This highlights an important trade-off: conservative rejection can preserve data quality, whereas end-to-end learning-based models may offer broader coverage but risk occasional mislabeling. Incorporating confidence thresholds or hybrid rule-learning mechanisms could help balance these approaches in future work.

SurveyNet’s strongest performance came from the BinaryMark subset, where it achieved 97.0% accuracy on gender classification and 88.7% accuracy when evaluating combined binary questions. The relative simplicity of the binary classification task, combined with clean visual separation between response zones, contributed to these high scores. Notably, this strong performance held even when multiple binary tasks were merged into a single model, illustrating SurveyNet’s flexibility and modularity for real-world survey deployments with varied binary fields. Performance dropped to 82.0% on the Yes/No dataset (3Y), where greater blank incidence and class imbalance reduced model reliability.

### 7.2. Persistent Challenges in DigitEntry Recognition

In contrast, the DigitEntry subset posed the greatest challenge. The model achieved only 46% accuracy with 20 training epochs (E20), improving slightly to 48% with 30 epochs (E30), both using the imbalanced dataset. This suggests that extending training time alone is not sufficient to overcome the limitations of sparse or skewed data. It was not until we introduced a balanced dataset (4Balanced) and extended training to 40 epochs with a smaller batch size that performance stabilized around 50% accuracy, with macro precision and recall also reaching 50%. Despite these modest gains, metric reports revealed substantial confusion among visually similar digits such as 3, 4, and 5, which likely stems from a combination of intrinsic visual similarity in handwritten markings and the limited variability of the training set.

To evaluate the significance of these improvements, we performed a Wilcoxon signed-rank test on per-class F1 scores comparing the E30 and 4Balanced configurations. The results were significant at the *p* = 0.041 level, indicating that even modest gains in accuracy reflect meaningful model improvements in this low-data, high-variance setting. These outcomes reflect the inherent complexity of recognizing handwritten digits in a low-data setting, SurveySet included only 135 DigitEntry images, distributed across five classes. Handwriting variability, limited stroke contrast, and inconsistent penmanship all contributed to the reduced performance. Compared to larger and more uniform handwriting datasets such as MNIST or IAM, SurveySet’s DigitEntry subset is both smaller and more heterogeneous, which constrains the model’s capacity to generalize.

A targeted noise-aware analysis on the DigitEntry subset further clarifies the role of data quality in recognition errors. Because handwritten digits rely on fine-grained stroke structure, they are especially sensitive to noise, blur, and stroke overlap, which can also introduce ambiguity between digits and marking strokes (e.g., “1” versus a slash). The observed monotonic decline in accuracy with increasing noise severity indicates that a substantial portion of DigitEntry errors is attributable to visual degradation rather than class imbalance or model architecture alone. This finding reinforces the importance of explicitly accounting for data quality when interpreting recognition performance on real-world handwritten inputs.

### 7.3. Implications of a Unified OCR-OMR Framework

Importantly, these results validate SurveyNet’s design choice to cast both OCR and OMR problems as a shared multi-class classification task. While the current implementation still relies on manual localization of response fields, the unified modeling approach removes the need for task-specific pipelines or template-dependent routing once the input region is extracted. In other words, all response types are processed using the same model weights and inference logic, eliminating the requirement for separate OCR or OMR modules at the classification stage. Future work will integrate automatic field detection and layout parsing to achieve full end-to-end automation. This unified inference design simplifies deployment and supports adaptability to varied survey formats. While the model employs a unified architecture for OCR and OMR tasks, the current study does not explicitly quantify cross-task knowledge transfer or shared feature learning, which remains an important direction for future investigation.

To further assess architectural compatibility with clean handwritten data, we evaluated the model on the MNIST dataset without additional fine-tuning. SurveyNet achieved an accuracy of 97.4% on MNIST, indicating that the observed limitations on SurveySet are driven primarily by dataset-specific noise and scarcity rather than architectural constraints alone. While SurveyNet still outperformed baseline OCR-only models on this task, future work could benefit from pretraining on handwriting datasets or incorporating localized attention to capture fine-grained stroke patterns.

### 7.4. Overall Interpretation

In summary, SurveyNet achieved up to 97.0% accuracy on binary tasks, 89.8% on multi-class scale recognition, and 84.0% on text-embedded multiple-choice fields, while maintaining a unified architecture capable of handling handwritten digits, symbolic marks, and informal responses with minimal manual intervention. Compared to updated benchmark models, SurveyNet consistently outperformed task-specific baselines on OMR tasks, exceeding OMRNet and classical methods by 4–10% in accuracy and F1 score across both structured and visually complex layouts. Although it underperformed on handwritten digit entry relative to a dedicated OCR CNN baseline (50.0% vs. 80–82% accuracy), this gap is largely attributable to limited and imbalanced training data. These results indicate that a single CNN-based framework can serve as a promising and scalable foundation for complex survey digitization workflows, while highlighting the need for task-aware modeling and expanded datasets to ensure consistent performance across response types.

## 8. Conclusions

This paper presents SurveyNet, a unified deep learning framework for automated digitization of survey responses. Unlike traditional systems that separate OMR and OCR into distinct pipelines, SurveyNet consolidates both within a single convolutional neural network architecture. As a reminder, the central contribution of this work is the unification of OCR and OMR tasks within one model, allowing consistent handling of different response types. All experiments in this work are conducted on a single survey template using fixed 28 × 28 input resolution and semi-automated region extraction. Consequently, the reported performance reflects task-specific behavior within this constrained setting and should not be interpreted as evidence of broad generalization across unseen questionnaire designs or layouts.

Experimental results show that SurveyNet achieves high classification accuracy across diverse input types, including up to 97.0% on binary responses, 89.8% on balanced Likert-scale recognition (ScaleMark), and 84.0% on text-embedded multiple-choice questions (OverlayMark). Even in challenging settings like handwritten digit entry, the model achieved 50.0% accuracy under balanced conditions, suggesting viability despite limited data. However, the model was trained and tested on a limited set of SurveySet forms, which may not reflect the full diversity of real-world questionnaires with different structures and visual designs. In particular, the significant deterioration of results in the DigitEntry subset indicates an insufficient number of handwritten digit samples, highlighting the need for a more comprehensive and representative training corpus. The model’s unified formulation eliminates the need for template-specific logic, supporting flexible deployment in noisy or unstructured survey environments.

SurveyNet’s main finding is that a single unified model can handle both OCR and OMR survey tasks, but performance depends strongly on response type. It achieved its best results on BinaryMark (up to 97%), followed by ScaleMark (89.8%) and OverlayMark (84%) when trained with balanced data, while DigitEntry remained the most difficult task at 50% accuracy due to limited data, handwriting variability, and noise. Overall, the results show that SurveyNet is effective for real-world survey digitization, especially when class balance and data quality are improved.

SurveyNet’s performance confirms that a shared classification strategy can successfully handle heterogeneous survey inputs within a fixed questionnaire template, while exhibiting task-dependent variability that reflects differences in data quality, visual complexity, and sample availability. These results lay the groundwork for more scalable and user-friendly digitization workflows in sectors such as healthcare, education, public services, and consumer research.

## 9. Future Work

Future work will explore expanding the dataset, incorporating handwriting-specific pretraining, and enabling full-page form interpretation by integrating layout detection and response zone segmentation. Additionally, we plan to incorporate model interpretability methods such as saliency mapping and attention visualization to make the model’s decision-making process more transparent and reliable for deployment in critical application domains such as healthcare and sociological research.

While the proposed model is lightweight and suitable for practical deployment, the current study does not provide a detailed evaluation of scalability or computational performance in large-scale or resource-constrained environments, which remains an important direction for future work. Future work will also focus on incorporating layout detection and response region localization to enable a fully end-to-end pipeline, along with expanding the experimental comparison to include a broader set of hybrid models for a more comprehensive evaluation.

We also recognize that while the model performs strongly on structured marks, it struggles with ambiguous or low-quality markings (e.g., partial ticks, faint strokes). Future work will include strategies to better characterize such failure cases and assess the impact of scan quality, including potential noise quantification for all the categories, to provide deeper context for performance variability. Furthermore, we aim to investigate hybrid strategies that combine traditional rule-based approaches with deep learning models to enhance robustness in challenging real-world scenarios.

Finally, a dedicated evaluation of digit–mark overlap will require explicit annotation of overlapping cases and construction of targeted test splits that isolate this failure mode from other sources of degradation, which we leave as an important direction for future work. A plan to systematically assess how user-related factors (e.g., age, handwriting style, marking behavior) and scan quality (e.g., resolution, skew, noise) can be observed to determine classification accuracy. This analysis will help quantify their impact and guide the development of adaptive preprocessing and model calibration techniques to improve reliability in diverse deployment settings.

## Figures and Tables

**Table 1:** Comparison of selected datasets relevant to Optical Character Recognition (OCR), Optical Mark Recognition (OMR), and survey form digitization.

Dataset	Survey Type	Marking Types	Character Types	Ground Truth	Layout Variability	Form Context
MNIST Dataset [43]	Handwritten digits	None	Handwritten digits (0–9)	Digit class	Low	No
IAM Dataset [44]	Handwriting	None	Full English sentences	Transcriptions (word, line, level)	Moderate	No
Afifi et al. Dataset [13]	Multiple-choice answer sheets	Bubbles, boxes, crosses	None	Selected choice	Moderate	Yes
SurveySet (Ours)	Customer experience surveys	Ticks, circles, crosses, bubbles	Handwritten digits	Mark class, digit class, question ID	High	Yes

**Table 2:** Overview of the SurveySet subsets used for unified OCR–OMR training. Each subset corresponds to a specific response type and label range, with representative examples and total image counts.

Subset	Response Type	Label Range	Examples	Image Count
ScaleMark	OMR (Likert scale)	1 to 7	Ticks, circles, slashes	2050
OverlayMark	OMR (text-overlaid)	1 to 4/1 to 5	Multiple-choice with printed labels	405
BinaryMark	OMR (binary options)	1 to 2	Yes/No, Male/Female	270
DigitEntry	OCR (handwritten digits)	1 to 5	Numeric input (e.g., vehicle count)	135

**Table 3:** Performance comparison of baseline models on SurveySet. Accuracy ranges reflect variation across dataset subsets (ScaleMark, OverlayMark, BinaryMark, DigitEntry), while Precision, Recall, and F1-score ranges correspond to per-class variability.

Model	Accuracy	Precision	Recall	F1 Score
Custom CNN [9]	80 to 82%	75 to 80%	75 to 76%	77 to 80%
OMRNet [12]	77 to 80%	77 to 79%	78 to 80%	75 to 78%
SOMR [40]	70 to 74%	69 to 75%	70 to 71%	65 to 66%
SurveyNet (Ours)	50 to 97%	52 to 96%	50 to 95%	51 to 96%

**Table 4:** Summary of precision, recall, F1-score for each class. The model achieved a macro-averaged F1 score of 0.90, with class-level F1 scores ranging from 0.84 to 0.95.

Class	Likert Label	Precision	Recall	F1 Score
1	Strongly Disagree	0.94	0.92	0.93
2	Disagree	0.95	0.91	0.93
3	Somewhat Disagree	0.93	0.97	0.95
4	Neutral	0.86	0.83	0.84
5	Somewhat Agree	0.90	0.90	0.90
6	Agree	0.88	0.86	0.87
7	Strongly Agree	0.81	0.92	0.86

**Table 5:** Summary of accuracy, precision, and recall across response categories, showing the highest performance for 2Balanced and the lowest for 2E.

Metrics	2E	2I	2M	2I + 2M + 2E	2Balanced
Accuracy	0.48	0.57	0.66	0.67	0.84
Precision	0.50	0.58	0.65	0.69	0.85
Recall	0.46	0.54	0.64	0.65	0.84

**Table 6:** Per-class precision, recall, and F1-score for the five ordinal choices. The model achieved a macro-averaged F1 score of 0.85, with class-level F1 scores ranging from 0.73 to 0.97.

Class	Ordinal Choice	Precision	Recall	F1 Score
1	Option 1	0.82	0.77	0.80
2	Option 2	0.69	0.79	0.73
3	Option 3	0.88	0.72	0.79
4	Option 4	0.91	1.00	0.95
5	Option 5	0.95	1.00	0.97

**Table 7:** A summary of accuracy, precision, and recall for 3G, 3Y, and 3G + 3Y. Performance is highest for 3G (0.97 across all three metrics), followed by 3G + 3Y (0.89), and lowest for 3Y (0.82).

Metrics	3G	3Y	3G + 3Y
Accuracy	0.97	0.82	0.89
Precision	0.97	0.82	0.89
Recall	0.97	0.82	0.89

**Table 8:** Per-subset performance metrics (accuracy, precision, recall, F1-score) for models trained separately on DigitEntry subsets.

Metrics	E20	E30	4Balanced
Accuracy	0.46	0.48	0.50
Precision	0.46	0.52	0.50
Recall	0.36	0.43	0.50

**Table 9:** A per-class precision, recall, and F1-score. The model achieved a macro-averaged F1 score of 0.63 and overall accuracy of 50%.

Class	Digit	Precision	Recall	F1 Score
1	Digit “1”	0.62	0.42	0.50
2	Digit “2”	0.24	0.73	0.36
3	Digit “3”	0.58	0.50	0.54
4	Digit “4”	0.90	0.47	0.62
5	Digit “5”	0.95	1.00	0.97

## Data Availability

The created deep learning framework, SurveyNet, is publicly available and accessible at https://github.com/mesreeja123/Survex (accessed on 16 June 2025). The SurveySet dataset presented in this study are openly available in Zenodo at https://doi.org/10.5281/zenodo.15610454 (accessed on 6 June 2025).
